# A Textbook of Protozoölogy

**Published:** 1909-12

**Authors:** 


**A Textbook of Protozoölogy.** By **Gary N. Calkins,** Ph.D., Professor of Protozoölogy in Columbia University, New York. Octavo, 349 pages, with 125 engravings and 4 colored plates. Lea & Febiger, Philadelphia and New York, 1909. (Cloth, $3.25 net.)

This compact and well written book presents an exhaustive study by a recognised authority on the protozoa,—the lowest class of germs in the animal kingdom, comprising simple cells, or colonies of cells,—which possess no nervous system and no circulatory organs. Within recent years the study of the lower forms of animal life in relation to disease, has been diligently pursued and evidence is constantly accumulating in favor of the view that certain diseases of animals and man are produced by protozoa. No previous exposition of the subject has equaled this one by Professor Calkins. J. A. R.

